# Genetic Structure Among 50 Species of the Northeastern Pacific Rocky Intertidal Community

**DOI:** 10.1371/journal.pone.0008594

**Published:** 2010-01-07

**Authors:** Ryan P. Kelly, Stephen R. Palumbi

**Affiliations:** Department of Biology, Hopkins Marine Station, Stanford University, Pacific Grove, California, United States of America; University of California San Diego, United States of America

## Abstract

Comparing many species' population genetic patterns across the same seascape can identify species with different levels of structure, and suggest hypotheses about the processes that cause such variation for species in the same ecosystem. This comparative approach helps focus on geographic barriers and selective or demographic processes that define genetic connectivity on an ecosystem scale, the understanding of which is particularly important for large-scale management efforts. Moreover, a multispecies dataset has great statistical advantages over single-species studies, lending explanatory power in an effort to uncover the mechanisms driving population structure. Here, we analyze a 50-species dataset of Pacific nearshore invertebrates with the aim of discovering the most influential structuring factors along the Pacific coast of North America. We collected cytochrome *c* oxidase I (COI) mtDNA data from populations of 34 species of marine invertebrates sampled coarsely at four coastal locations in California, Oregon, and Alaska, and added published data from 16 additional species. All nine species with non-pelagic development have strong genetic structure. For the 41 species with pelagic development, 13 show significant genetic differentiation, nine of which show striking F_ST_ levels of 0.1–0.6. Finer scale geographic investigations show unexpected regional patterns of genetic change near Cape Mendocino in northern California for five of the six species tested. The region between Oregon and Alaska is a second focus of intraspecific genetic change, showing differentiation in half the species tested. Across regions, strong genetic subdivision occurs more often than expected in mid-to-high intertidal species, a result that may reflect reduced gene flow due to natural selection along coastal environmental gradients. Finally, the results highlight the importance of making primary research accessible to policymakers, as unexpected barriers to marine dispersal break the coast into separate demographic zones that may require their own management plans.

## Introduction

Uncovering mechanisms that determine gene flow is critical for understanding population ecology, the scale of natural selection across environmental gradients, and decisions about sustainable exploitation. This is especially true where conservation strategies emphasize the creation of management zones such as wildlife parks or marine protected areas [see 1]. The genetics of species across these management mosaics have long been a part of conservation biology and molecular ecology [2]. Recent emphasis, especially in the sea, on ecosystem-based management as a main goal for sustainable use of natural areas [3], [4] indicates that knowing the population structure of single species is no longer enough. Instead, understanding the population genetic patterns and the processes that create them for a wide set of species within a habitat has become an important part of the goal [5].

Such data are particularly relevant in marine ecosystems because of the possibility that many species have wide dispersal [6], and the increasing focus on marine protected areas as a management strategy [1]. Because these areas are usually too small to contain self-seeding populations of most high dispersal marine species [7], networks of protected areas connected by dispersal are often required [8], [9], [10]. Planktonic duration of dispersing larvae is often used as a proxy for dispersal potential [see, e.g., 10] and is used in management decisions about marine resources. Comparing population genetic patterns across the same seascape for many species can allow initial identification of species with different levels of structure, and test hypotheses about the processes that create dispersal variation for species in the same ecosystem.

Uncovering common causes of genetic subdivision across a shared landscape requires a synthesis of both genetic and ecological information from a diverse array of species [11]. Recent efforts have focused on fine scale landscape or seascape genetic tests of the importance of geographic features in determining gene flow patterns [see 12]. However, it is not necessarily the case that all species, even with similar life histories, will react the same way to the same geography. Landscape or seascape genetic studies, if conducted for many species in a controlled way with similar genetic tools, can have the statistical power to detect the impact of particular geographic features or life history traits on genetic structure.

In the marine environment, much of our thinking about genetic connectivity has focused on two major factors: mode of larval development and biogeographic barriers. Case studies of marine species pairs with different life histories [e.g.], [13], [14], [15], [16,17] have demonstrated that species with no pelagic larval dispersal (i.e., brooding or viviparous species, or those with demersal egg sacs and no subsequent dispersal stage) tend be highly subdivided, suggesting very low gene flow among these populations [8]. This result has been often generalized to suggest that longer pelagic durations will have greater gene flow. Longer pelagic development is expected to result in greater dispersal, lower genetic differentiation and better-connected populations [18], [19]. However, some striking exceptions have been well studied, such as the tide pool copepod *Tigriopus californicus* that has remarkably short scale population structure [20], and a suite of Caribbean marine fish for which larval duration explains little of the variation in mtDNA differentiation [21]. As a result, there remains a large and unexplained variability in levels of genetic structure among ecologically similar, pelagically-dispersing invertebrate species [16], [17], [22]

In some cases, genetic differentiation appears ruled by major biogeographic breaks rather than larval biology [23], [24]. However, not all biogeographic breaks are associated with strong genetic differentiation [25]. The strong biogeographic break in California at Pt. Conception, for example, is home to a few well-known shifts in marine population genetics [26], [27], but many species show little or no divergence in the region [see 25], [28], [29].

Here, we present original mtDNA data from 34 invertebrate species from the nearshore environment of the Pacific coast of North America. Combining this dataset with published data for 16 additional species across the same geographic area, we use a multiple lineage regression and ANOVA framework to ask whether levels of genetic differentiation vary significantly with pelagic duration and presence of biogeographic barriers. We find that, consistent with earlier work, species without pelagic larval dispersal have significantly more subdivision among populations. However our analysis of the relationship between genetic structure and a suite of species and habitat traits shows that increased pelagic duration has little power to explain genetic subdivision. Instead, significant variation in genetic structure among these 50 species is explained by adult habitat depth, with high-to-mid intertidal species of many taxonomic lineages showing strong genetic structure. In addition, we find that the upwelling center of Cape Mendocino, historically neglected by genetic sampling, houses a number of interesting genetic shifts even for high dispersal species.

## Results

### Genetic Structure among species

**Figure 1:**
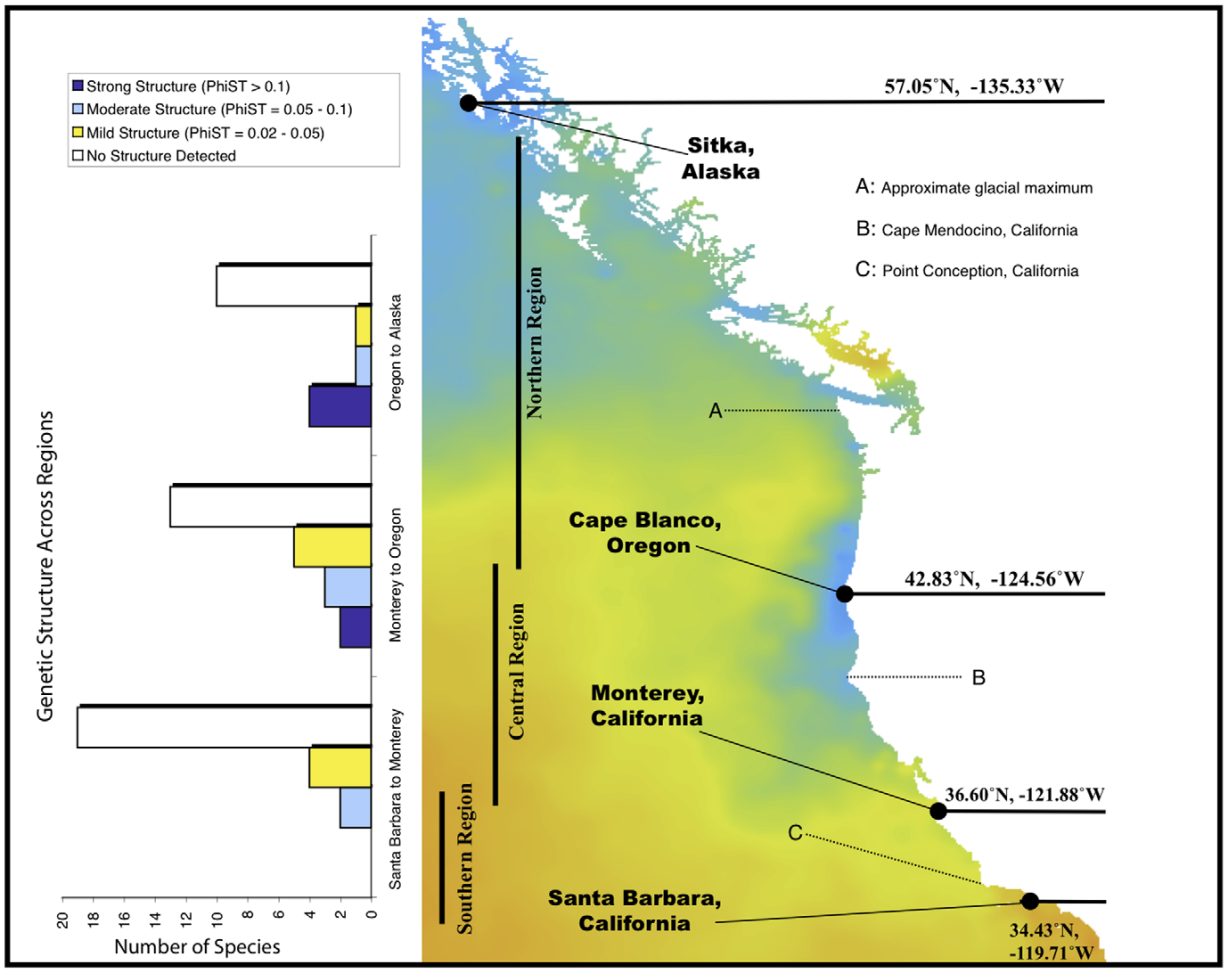
Map of primary collection locations, geographic regions, and major ecological features of the Pacific coast of North America. The background color represents sea surface temperature for a day in July, 2008. Bar graphs on the left-hand side of the figure show the number of species with strong, moderate and mild genetic structure in southern, central and northern comparisons. Structure categories are mild: Φ_ST_ = 0.02 – 0.05, moderate: 0.05 – 0.10, strong:>0.1.

We sampled 34 nearshore invertebrate species across four locations on the Pacific coast of North America (Sitka, Alaska; Cape Blanco, Oregon; Monterey, California, and Santa Barbara, California), and added data from 16 species for which published data were available. Of these 16, 12 used COI data (Table 1). Forty-nine of 50 species (all but the sea anemone *A. elegantissima*) showed intraspecific variation in COI sequence. Across this set, all nine species with non-pelagic development showed significant genetic structure (mean Φ_ST_ = 0.53, range = 0.2–1.0, Table 2, and Supplementary Information). We included the harpactacoid tidepool copepod *Tigriopus californicus* in this list because of its largely benthic habits. Among 41 species with pelagic larvae, we found genetic differentiation in 13 (32%). There was strong structure (Φ_ST_ = 0.11 – 0.6; p<0.001) in nine species, and moderate structure (Φ_ST_ 0.02 – 0.10; p<0.05) in four more (Figure 1).

**Table 1:** Species, collection locations, and gene fragment information.

Species		Collection Data					Molecular Data			
		Santa Barbara	Monterey	Oregon	Alaska	Other	Gene Fragment	Fragment Length Analyzed (bp)	Primer Set	Annealing Temp (C)
Annelida	*Phragmatopoma californica*	16	18	–	–	–	COI	ca. 550	LCO1490-HCO2198 (Folmer et al. 1994)	52
Arthropoda	*Balanus glandula*	48	56	46	–	–	COI	See Sotka et al. 2004		
	*Cancer antennarius*	20	13	8	–	–	COI	ca. 550	LCO1490-HCO2198 (Folmer et al. 1994)	52
	*Cancer productus*	4	4	9	2	–	COI	ca. 550	LCO1490-HCO2198 (Folmer et al. 1994)	52
	*Emerita analoga*	24	17	22	–	–	COI	ca. 550	LCO1490-HCO2198 (Folmer et al. 1994)	52
	Hemigrapsus nudus	–	14	24	9	Russian Gulch St. Pk. (10)	COI	ca. 550	LCO1490-HCO2198 (Folmer et al. 1994)	52
	*Idotea cf. stenops*	16	–	–	–	Pigeon Pt. (11)	COI	240	IdoCOIf-r (T. Bell and J. Wares, pers comm.)	48
	*Idotea kirchanskii*	–	65	–	–	Leo Carrillo St. Bch. (17)	COI	240	IdoCOIf-r (T. Bell and J. Wares, pers comm.)	48
	*Idotea montereyensis*	–	–	41	24	Pigeon Pt. (17)	COI	240	IdoCOIf-r (T. Bell and J. Wares, pers comm.)	48
	*Lophopanopeus bellus*	–	8	10	25	–	COI	ca. 550	LCO1490-HCO2198 (Folmer et al. 1994)	52
	*Pachygrapsus crassipes*	–	–	73	–	San Louis Opispo (20)	COI	See Cassone and Boulding 2006		
	*Pagurus granosimanus*	–	12	15	8	Cape Viz. (17), Pigeon Pt. (13), Pt. Fermin (27)	12S-tRNA-COI	ca. 700	12air-HCO2198 (Cunningham, pers comm., and Folmer et al. 1994)	55
	*Pagurus hirsutiusculus*	–	–	20	8	Patrick's Pt. (21), Cape Mendocino (13), Cape Vizcaino (17), San Francisco (8), Pigeon Pt. (19)	12S-tRNA-COI	ca. 700	12air-HCO2198 (Cunningham, pers comm., and Folmer et al. 1994)	55
	*Pagurus samuelis*	–	39	20	–	Patrick's Pt. (9), Cape Vizcaino (15), Fitzgerald Marine Reserve (8), Jade Cove (6), Pt.Fermin (10)	12S-tRNA-COI	ca. 700	12air-HCO2198 (Cunningham, pers comm., and Folmer et al. 1994)	55
	*Pagurus venturensis*	22	–	–	–	Morro Bay (16)	12S-tRNA-COI	ca. 700	12air-HCO2198 (Cunningham, pers comm., and Folmer et al. 1994)	55
	*Pandalus platyceros*	25	17	–	–	–	COI	ca. 550	Crustacean COI Fwd - HCO2198 (Costa et al. 2007, Folmer et al. 1994)	48
	*Petrolisthes cinctipes*	–	22	11	–	–	AT-Rich Region	ca. 700	12Srev-COIr3 (Cunningham, pers comm)	55
	*Pollicipes polymerus*	14	14	26	12	Patrick's Pt. (22), Sinkyone Wilderness (19)	COI	ca. 550	LCO1490-HCO2198 (Folmer et al. 1994)	52
	*Pugettia gracilis*	–	–	8	14	–	COI	ca. 550	LCO1490-HCO2198 (Folmer et al. 1994)	52
	*Semibalanus cariosus*	–	12	18	10	–	COI	ca. 550	LCO1490-HCO2198 (Folmer et al. 1994)	52
	*Tetraclita squamosa*	56	84	–	–	–	4 allozymes	See Ford and Mitton 1993		
	*Tigriopus californicus*	5	4	–	–	(Fst estimated from tree diagrams)	COI, nuclear H1	See Burton 1998, Burton and Lee 1994		
Cnidaria	*Anthopleura elegantissima*	17	18	9	–	–	COI	ca. 550	LCO1490-HCO2198 (Folmer et al. 1994)	52
Echinodermata	*Cucumaria pseudocurata*	–	–	27	–	Pecsadero Pt. (25)	mtDNA control region	See Arndt and Smith, 1998		
	*Parastichopus parvimensis*	34	40	–	–	–	COI	470	LCO1490-HCO2198 (Folmer et al. 1994)	52
	*Pisaster giganteus*	18	29	–	–	–	COI	450	Pat2-Eco1 (Knott and Wray 2000)	52
	*Pisaster ochraceus*	–	15	10	–	–	COI	See Harley et al. 2006		
	*Pycnopodia helianthoides*	24	19	4	23	–	COI	450	Pat2-Eco1 (Knott and Wray 2000)	52
	*Strongylocentrotus franciscanus*	23	27	–	21	–	COI	450	Pat2-Eco1 (Knott and Wray 2000)	50
Mollusca	*Acanthina spirata*	9	6	–	–	(Fst estimated from spanning network)	COI	See Hellberg et al 2001		
	*Alderia modesta*	–	–	12	12	San Francisco (16)	COI	See Ellingson and Krug 2006		
	*Alderia willowi*	–	–	–	–	San Francsico (32), San Pedro (14)	COI	See Ellingson and Krug 2006		
	*Aplysia californica*	39	39	–	–	–	COI	See Medina and Walsh 2000		
	*Calliostoma ligatum*	–	18	25	11	–	COI	ca. 550	LCO1490-HCO2198 (Folmer et al. 1994)	52
	*Cyanoplax dentiens*	–	13	–	–	Eagle Cove (9)	COI	See Kelly and Eernisse 2007		
	*Fissurella volcano*	20	17	–	–	–	COI	450	Lottia COI fwd- HCO2198 (this paper, Folmer et al. 1994)	50
	*Haliotis rufescens*	46	24	–	–	Crescent City (31)	COI, nuclear microsats, AFLP	See Gruenthal et al. 2007		
	*Katharina tunicata*	–	10	19	13	–	COI	ca. 550	LCO1490-HCO2198 (Folmer et al. 1994)	52
	*Lottia austrodigitalis*	22	23	–	–	–	COI	ca. 600	COIH-di- COIL-di (D. Eernisse, pers comm)	51
	*Lottia digitalis*	–	–	23	15	Cape Mendocino (10), Mendocino (10), Fitzgerald Marine Reserve (19), Pigeon Point (10)	COI	ca. 600	COIH-di- COIL-di (D. Eernisse, pers comm)	51
	*Lottia paradigitalis*	15	18	22	9	–	COI	450	Lottia COI fwd- HCO2198 (this paper, Folmer et al. 1994)	51
	*Lottia pelta*	–	20	10	14	–	COI	400	LCO1490-Lottia COIrev (this paper, Folmer et al. 1994)	51
	*Lottia sp. nov., cf. pelta*	18	30	–	–	–	COI	450	LCO1490-Lottia COIrev (this paper, Folmer et al. 1994)	51
	*Macoma nasuta*	14	24	21	–	-	COIII	540	MaCO3f-MaCO3r (Nikula 2007)	55
	*Mytilus californianus*	–	85	85	–	(values represent means for sampled loci)	8 mtDNA regions	See Ort and Pogson 2007		
	*Nucella emarginata*	25	25	–	–	(Fst estimated from tree diagrams)	COI, 12S	See Marko 1998		
	*Nucella ostrina*	–	25	25	25	(Fst estimated from tree diagrams)	COI, 12S	See Marko 1998, 2004		
	*Olivella biplicata*	22	15	23	–	–	COI	ca. 550	LCO1490-HCO2198 (Folmer et al. 1994)	52
	*Searlesia dira*	–	–	11	9	–	COI	ca. 550	LCO1490-HCO2198 (Folmer et al. 1994)	52
	*Tegula funebralis*	20	29	22	–	–	COI	ca. 550	LCO1490-HCO2198 (Folmer et al. 1994)	52

The numbers of individuals collected for each species at each focal collection location (Sitka, Cape Blanco, Monterey, Santa Barbara; see text) are shown, along with collections made at alternative locations or for supplementary population-level analysis. We designed two novel primers to amplify and sequence cytochrome *c* oxidase, subunit I (COI) in limpets; their sequences were as follows (5′– 3′): Lottia COI fwd: TTTATAGTNATGCCAGTATTAATTGG; Lottia COI rev: CTAGCGAARATNGAAGCAATTCC. Unpublished primers were provided courtesy of C. Cunningham (Duke University), T. Bell and J. Wares (University of Georgia), and D. Eernisse (California State University, Fullerton) as noted; published primer sources are cited. In most cases, the COI fragment was used for analysis; in some species amplification of this fragment was problematic due to the presence of pseudogenes or other complications. In these cases, we amplified a fragment that spanned one or more gene boundaries, resulting in a single fragment. DNA sequence data are available online in Genbank.

**Table 2:** A partial list of ecological and life history information used for analysis.

Species	Phylum	Min. PLD	Tidal Height (4 categories)	COI Nucleotide Diversity	PhiST	SB-Mont FST	Mont-OR FST	OR-AK FST
*Phragmatopoma californica*	Annelida	18	mid	0.01	0.01978	0.01978	NA	NA
*Balanus glandula*	Arthropoda	14	high	NA	NA	**0.043**	**0.329**	NA
*Cancer antennarius*	Arthropoda	60	low	NA	NA	NA	NA	NA
*Cancer productus*	Arthropoda	100	low	NA	NA	NA	NA	NA
*Emerita analoga*	Arthropoda	70	low	0	−0.0151	−0.00713	−0.01681	NA
*Hemigrapsus nudus*	Arthropoda	30	high	0.01	**0.438**	NA	**0.02432**	**0.595**
*Idotea cf. stenops*	Arthropoda	0	low	0	**0.861**	**0.8607**	NA	NA
*Idotea kirchanskii*	Arthropoda	0	low	0.01	**0.145**	**0.14527**	NA	NA
*Idotea montereyensis*	Arthropoda	0	low	0.01	**0.087**	NA	**0.13337**	−0.01259
*Lophopanopeus bellus*	Arthropoda	30	low	0.01	0.00727	NA	0.03239	0.00154
*Pachygrapsus crassipes*	Arthropoda	30	high	0.01	NA	0.0034	−0.00055	−0.00055
*Pagurus granosimanus*	Arthropoda	70	mid	0.01	**0.103**	−0.00797	**0.04453**	**0.5**
*Pagurus hirsutiusculus*	Arthropoda	67	mid	0	**0.357**	NA	**0.08365**	**0.833**
*Pagurus samuelis*	Arthropoda	51	mid	0.01	**0.104**	**0.11933**	**0.04867**	NA
*Pagurus venturensis*	Arthropoda	50	mid	0.01	**0.252**	NA	NA	NA
*Pandalus platyceros*	Arthropoda	150	deep water	0	−0.0062	−0.00616	NA	NA
*Petrolisthes cinctipes*	Arthropoda	30	mid		0.01456	NA	0.01456	NA
*Pollicipes polymerus*	Arthropoda	42	mid	0.01	**0.034**	**0.09881**	**0.05929**	0.03261
*Pugettia gracilis*	Arthropoda	120	low	0	0.00252	NA	NA	0.00252
*Semibalanus cariosus*	Arthropoda	90	mid	0	−0.0006	NA	0.00657	0.00081
*Tetraclita squamosa*	Arthropoda	20	high	NA	0.011	0.011	NA	NA
*Tigriopus californicus*	Arthropoda	28	high	NA	**0.98**	**0.98**	**0.98**	**0.98**
*Anthopleura elegantissima*	Cnidaria	30	mid	0	0	NA	NA	NA
*Cucumaria pseudocurata*	Echinodermata	0	low	NA	**0.5**	NA	**0.5**	NA
*Parastichopus parvimensis*	Echinodermata	50	low	NA	0.0001	0.0001	NA	NA
*Pisaster giganteus*	Echinodermata	60	low	0	−0.0261	−0.02613	NA	NA
*Pisaster ochraceus*	Echinodermata	76	mid	0	NA	0	**0.03**	NA
*Pycnopodia helianthoides*	Echinodermata	70	low	0	**0.137**	0.00353	NA	−0.07797
*Strongylocentrotus franciscanus*	Echinodermata	70	low	0	0.009	0.03155	NA	NA
*Acanthina spirata*	Mollusca	0	high	NA	**0.5**	0.5	NA	NA
*Alderia modesta*	Mollusca	35	low	0.02	0.00838	NA	−0.01151	−0.00143
*Alderia willowi*	Mollusca	2	low	0.01	0.01197	−0.00926	NA	NA
*Aplysia californica*	Mollusca	30	low	NA	0.0084	0.0084	NA	NA
*Calliostoma ligatum*	Mollusca	7	low	0	−0.009	NA	−0.00814	0.00192
*Cyanoplax dentiens*	Mollusca	6	mid	0.01	**0.039**	NA	**0.06542**	NA
*Fissurella volcano*	Mollusca	4	mid	0	0.01089	0.01089	NA	NA
*Haliotis rufescens*	Mollusca	4	low	NA	0.007	−0.008	0.017	NA
*Katharina tunicata*	Mollusca	7	mid	0.01	0.02932	NA	−0.00952	0.07578
*Lottia austrodigitalis*	Mollusca	5	high	0	−0.038	−0.03802	NA	NA
*Lottia digitalis*	Mollusca	5	high	0	**0.611**	NA	**0.64658**	0.01288
*Lottia new sp. cf pelta*	Mollusca	5	mid	0	−0.033	−0.03298	NA	NA
*Lottia paradigitalis*	Mollusca	5	mid	0	0.00055	0.0288	−0.03858	−0.03255
*Lottia pelta*	Mollusca	5	mid	0	**0.561**	NA	−0.00683	**0.673**
*Macoma nasuta*	Mollusca	35	low	NA	0.01147	−0.00616	0.0116	NA
*Mytilus californianus*	Mollusca	9	mid	NA	−0.001	0	0	0
*Nucella emarginata*	Mollusca	0	high	NA	**0.5**	**0.7**	NA	NA
*Nucella ostrina*	Mollusca	0	high	NA	**0.2**	NA	**0.7**	0
*Olivella biplicata*	Mollusca	1	low	0.01	0.00621	0.00025	0.01235	NA
*Lirabuccinum (Searlesia) dira*	Mollusca	0	mid	0	**1**	NA	NA	**1**
*Tegula (Chlorostoma) funebralis*	Mollusca	5	mid	0.01	0.01674	−0.00047	0.01276	NA

See Supplementary Information S1 for the complete dataset. Genetic results in bold are significantly different from zero. PLD  =  pelagic larval duration. Pairwise F_ST_ calculations are shown for pairs of populations: “Mont-OR,” for example, is the pairwise F_ST_ between Monterey and Oregon. SB  =  Santa Barbara, Mont  =  Monterey, OR  =  Oregon, AK  =  Alaska.

### Geography of genetic structure

A greater proportion of species had significant mtDNA differences in the northern and central regions than in the south. Between Oregon and Sitka, Alaska 33% of species showed structure (Figure 1). Between Monterey and Oregon, 40% showed mtDNA differentiation. By contrast, only 15% of species sampled between Monterey and Santa Barbara had genetic differentiation, and this was not strong for any of the species we sampled (Figure 1). Central comparisons have a greater fraction of high, moderate and mildly differentiated species than do southern comparisons, and the northern comparisons have the highest fraction of species with strong genetic structure (Figure 1). These results were uncorrelated with the geographic distance between neighboring sampled populations. Normalizing F_ST_ values for different geographic distances between sampling sites did not appreciably change their distribution.

Not every species occurs at each sampling site, resulting in a dataset with irregular sampling: only four species, for example, occur at all four sampling sites. However, the observed geographic trends are qualitatively evident in subsets of the data (species sampled at only two locations, for example, have a higher mean pairwise F_ST_ between Monterey and Oregon (0.0366; n = 3 spp) than between Santa Barbara and Monterey (−.0028; n = 11 spp).

**Figure 2:**
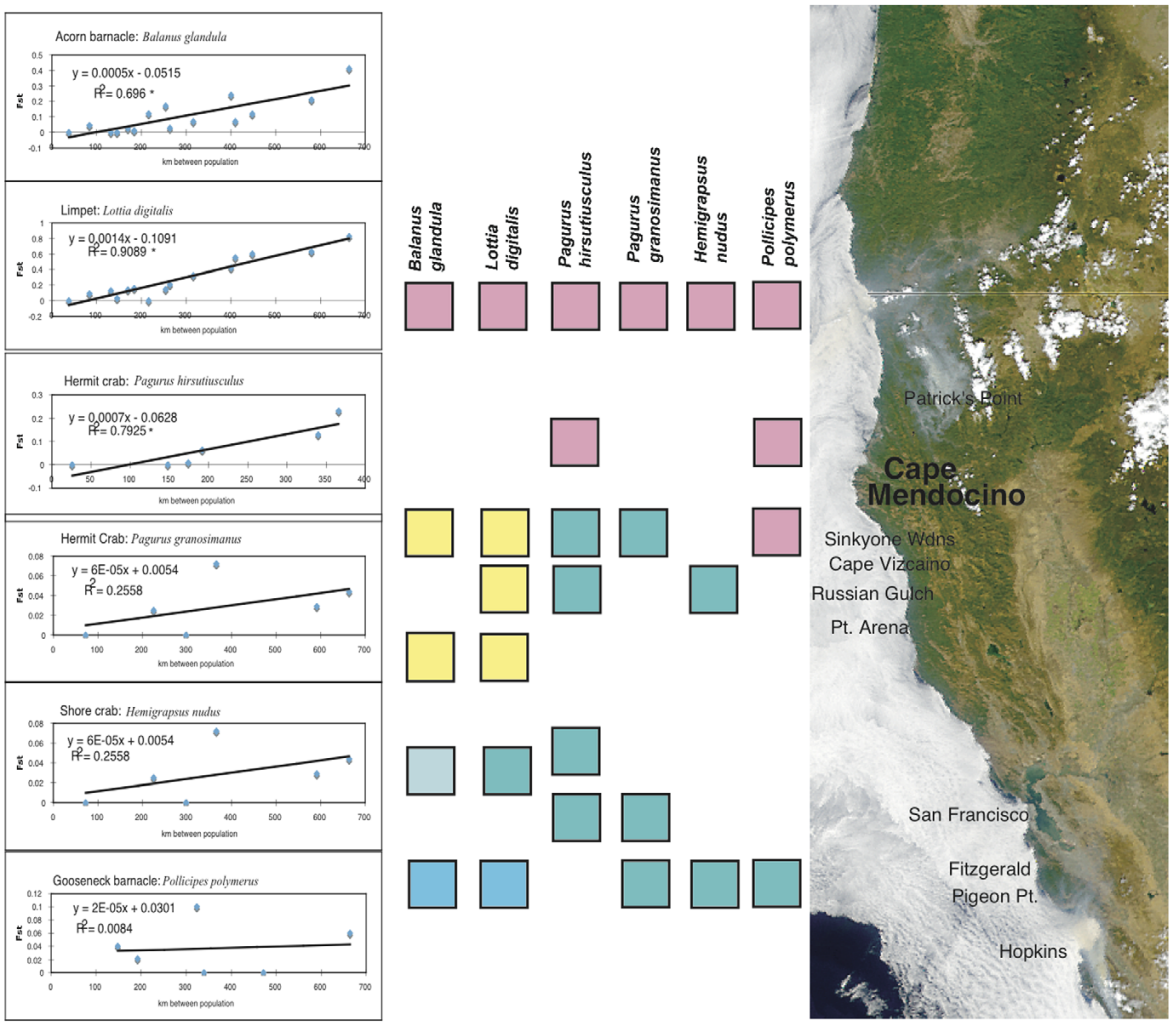
Genetic differentiation in the vicinity of Cape Mendocino, CA, for six invertebrate species. Squares represent sampling localities for each species (different habitat requirements precluded identical sampling sites). Squares with different shading are significantly different in Arlequin analyses based on COI haplotypes for each species. Sample sizes and exact collection locations are shown in Table 1. Inset is the correlation between geographic and genetic distance for the same six species, for collections made in the Cape Mendocino region. Asterisk denotes significance at the p = 0.05 level.

For six species that showed significant mtDNA differentiation across the central range (*Balanus glandula, Pagurus granosimanus, P. hirsutiusculus, Lottia digitalis, Pollicipes polymerus*, and *Hemigrapsus nudus*), we sampled intermediate locations to further determine the geographic pattern of genetic shifts. In five of six cases, Cape Mendocino emerges as an important phylogenetic feature, though several different patterns emerged for different species (Figure 2). The limpet *L. digitalis* and the barnacle *B. glandula* show broad genetic clines from Monterey to Cape Mendocino [see also 30], with strong isolation-by-distance signatures along the central California coast. For the hermit crabs *Pagurus hirsutiusculus* and *P. granosimanus*, populations in Monterey are genetically similar to those in northern California, but are differentiated from populations to the north of Cape Mendocino. For *P. hirsutiusculus*, this differentiation occurs abruptly between collections made in the Sinkyone Wilderness and at Patrick's Point (Figure 2). Among populations of the shore crab *Hemigrapsus nudus*, Sitka populations were the most differentiated. However, Oregon populations show a mtDNA haplotype shared with Alaska but not California, and populations are differentiated across Cape Mendocino. By contrast, the gooseneck barnacle *Pollicipes polymerus* is the only of these species that does not show genetic differentiation across Cape Mendocino. Current data show mild differentiation only of the Monterey population from others in the data set.

Isolation-by-distance patterns along the central coast of California are significant in three of six species (Mantel tests, Figure 2) - further sampling may reveal significant patterns in two others. Slopes ranged from 6×10^−5^ km^−1^ to 1.4×10^−3^km^−1^, a 20-fold range that indicates substantial difference in effective migration rate. The highest slopes were seen in *Lottia digitalis*, a limpet with larvae that spend only 5–10 days in the pelagic phase. However, the slope of the line for the hermit crab *P. hirsutiusculus* is only two-fold less, even though this species has a pelagic period lasting 60 days or more. Though more data on multiple loci are needed to provide a high resolution view of gene flow, these comparisons suggest that larval period is not the dominant structuring agent in these species.

### Correlates of genetic structure

**Figure 3:**
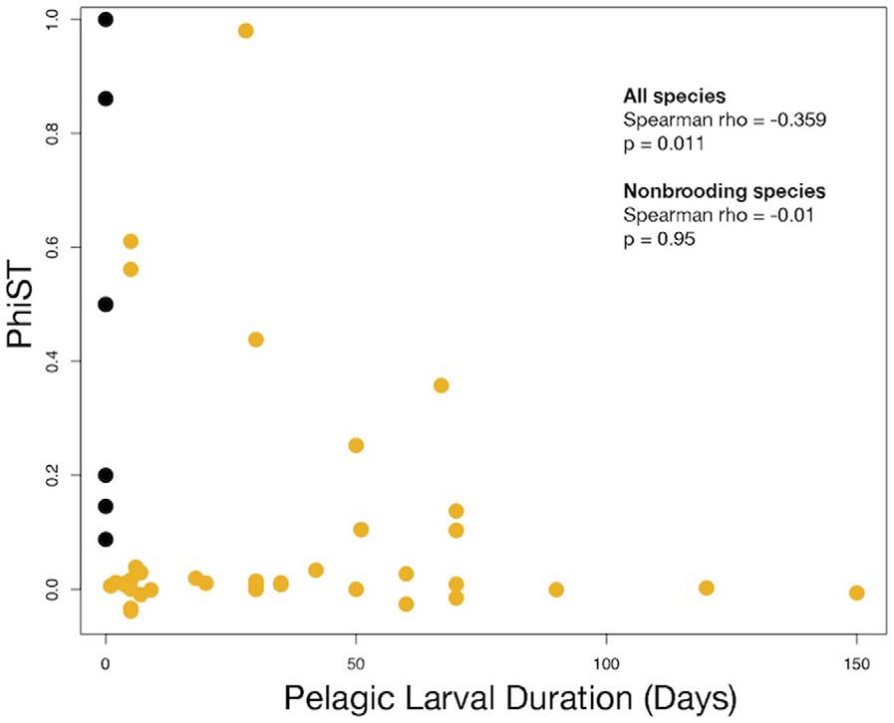
Overall genetic subdivision (Φ_ST_) and pelagic larval duration (PLD). Brooding species (nonpelagic larvae) in black filled circles, nonbrooding species (pelagic larvae) in orange filled circles. Statistical results for the nonparametric Spearman Rank Correlation are shown.

In the whole data set, larval pelagic period was strongly associated with Φ_ST_ values (p = 0.011), but this correlation disappeared when non-pelagic species were excluded from the analysis (rho = −0.01, p = 0.95, Figure 3). For example, long pelagic durations of 50–67 days were associated with Φ_ST_ values as high as 0.1 – 0.36 (Table 1).

**Figure 4:**
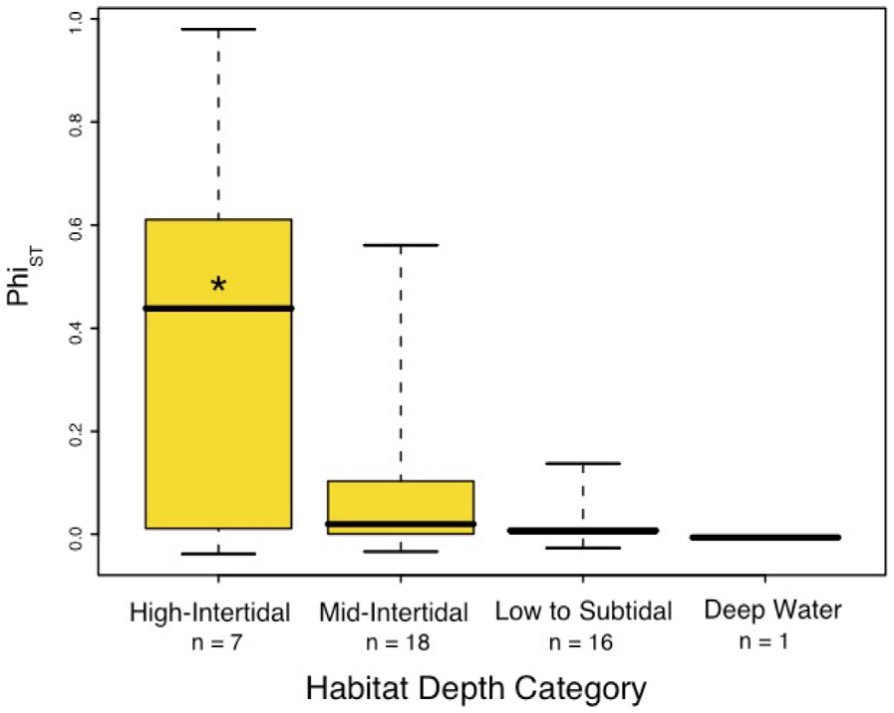
Overall genetic subdivision (Φ_ST_) and habitat depth for nonbrooding species for four habitat categories. Asterisk denotes significant the difference between the high intertidal depth category and both the mid-intertidal and low-subtidal categories (p<0.05). Box widths are 25^th^ and 75^th^ quartiles, respectively; bars are means, and whiskers are the extreme data points. Φ_ST_ values are significantly or marginally significantly apportioned among depth categories (ANOVA R^2^ = 0.24, p = 0.02). High and Mid-Intertidal species had significantly and marginally greater Φ_ST_ values than Low to Subtidal species, respectively (Wilcoxon, p = 0.03 and p = 0.07). Depth categories were sampled approximately equally in each geographic region (chi-square, all region pairs p>0.2).

We examined further potential correlates of genetic structure with a multiple linear regression that included Φ_ST_ and pairwise population F_ST_ as the dependent variables and 22 ecological, habitat, and molecular traits as possible independent variables (Table 2, and Supplementary Information). The strongest result was significant differentiation among habitat depth categories (ANOVA, R^2^ = 0.24; p = 0.02), with species occurring higher in the intertidal zone being significantly more subdivided than those at lower depths (Figure 4). This trend was robust to additional partitioning of habitat depth into different numbers of categories (four or seven), coded as either numeric or categorical variables (as shown in Figure 4). Strong taxonomic biases are present in the dataset as a result of nonrandom species sampling and the availability of data (see Table 2, and Supplementary Information). However the inverse relationship between genetic structure and habitat depth is not a product of taxonomic sampling bias. Subsampling the dataset to include a single species from either each taxonomic family (n** = **28) or each order (n = 15) demonstrated a high degree of phylogenetic independence, with the trend remaining significant in 91% (family) or 86% (order) of the subsampled dataset replicates. Neither nucleotide diversity nor Tajima's D meaningfully correlated with Φ_ST_, or with any pairwise F_ST_ calculation between sites.

## Discussion

About one in three sampled west coast invertebrate species with pelagic larvae shows genetic differentiation between southern California and Alaska. Comparison of similar data sets among many species in a similar geographic context allows tests of hypotheses about the factors associated with genetic structure and low gene flow. Our results highlight a little-studied area of the U.S. west coast as an important dispersal barrier and suggests a role for adult habitat in genetic differentiation.

Despite its importance as a site of environmental change and faunal turnover, we found little population genetic structure across Point Conception between Monterey and Santa Barbara. Genetic change in invertebrate species with pelagic larvae has been reported across Point Conception [25], [27], [29], and we add four invertebrate species to this list. Likewise, Sivasundar and Palumbi (submitted) in a meta-analysis of population structure of 15 species of west coast Sebastes rockfish report differentiation of five species at Point Conception. Yet, strong structure in these cases is rare [25], [27] except for estuarine species [29] or species with low dispersal potential. Rather, upwelling relaxation and El Niño events may transport pelagic larvae northward around Point Conception with some regularity [31], [32], while the California current may successfully carry northern larvae southward [27]. Such bi-directional gene flow likely prevents large scale genetic differentiation in many taxa.

A larger fraction of populations are differentiated across Cape Mendocino in northern California, and between Oregon and Alaska. For northern comparisons, our data cannot pinpoint the area of genetic change between Oregon and Sitka Alaska. However, there is a major bifurcation in oceanic currents near the entrance to Puget Sound, with some flow moving northward, and some forming the California Current heading south. This major current shift has been suggested to be responsible for genetic differences in a number of fish and invertebrate species [e.g., 33]. We found reciprocally monophyletic mtDNA clades or dramatic haplotype frequency differences in several species across the region (including *Lottia pelta*, *Hemigrapsus nudus*, *Pagurus hirsutiusculus*, *P. granosimanus*, and *Searlesia dira*); the resulting F_ST_ values were much higher in the northern region than elsewhere for the same species. These dramatic differences are in marked contrast to the mild differences at Point Conception, and could be the focus of future study. It may be that clade-level differences in the north, as opposed to mere allele frequency differences, are primarily driven by glacial cycling [16].

### Cape Mendocino as a barrier

Our data provide a finer look at the potential for genetic differentiation at Cape Mendocino. Among six species, five show differences across the Cape (Figure 2). However, different species have different patterns of genetic differentiation, from broad genetic clines to sharper genetic breaks. Isolation-by-distance patterns are strong for several species, but the slopes of the lines relating genetic and geographic distance vary widely (Figure 2).

Sotka et al. [30] previously showed a shift in barnacle gene frequencies at mtDNA and nuclear loci from Monterey to Oregon, probably due to a combination of dispersal and selection [34]. Our data on the limpet *Lottia digitalis* show a similar strong genetic cline over several hundred km. Inspection of intraspecific haplotype networks shows that there are two sets of related haplotypes differing by two base pairs in this species, and that these clades shift in relative abundance from northern to southern populations. Southern Oregon is dominated by one clade (>90%) whereas populations south of San Francisco show >90% the alternative clade. Further south, this limpet species is replaced by a cryptic species newly named *Lottia austrodigitalis* [35]. Shanks [36] showed that *L. digitalis* spawned during downwelling oceanographic conditions when offshore transport of larvae is likely to be limited. Such restricted realized larval dispersal may help to explain the strong genetic structure along the California coast in this species.

Other species with structure do not appear to have these obvious limits to dispersal potential. The hermit crabs *Pagurus hirsutiusculus* and *P. granosimanus* have large eggs and larval periods of 9–10 weeks [37]. Several species of nearshore fish also show strong genetic breaks at Cape Mendocino [38]. Long planktonic durations are associated in these species with low differentiation along the California coast between Monterey and Cape Mendocino (Figure 2). In these cases, genetic differences occur closer to Cape Mendocino itself and may be driven by offshore movement at Cape Mendocino of the south-flowing California Current. Surface drifters released from Oregon tend to veer offshore at this point [30]: strong upwelling jets and seasonal offshore currents [39], [40] may form a barrier that has a structuring effect on some marine populations in this area. This barrier is not impenetrable, however: many species, including some with low pelagic durations, have no discernable structure.

Broad geographic concordance of genetic structure among species has been used as a signal of the impact of geographically initiated dispersal boundaries on genetic differentiation [41]. For Cape Mendocino, our data show that multiple species exhibit genetic differentiation across this geographic feature. Unsurprisingly, patterns of genetic differentiation are not entirely concordant, suggesting that currents or other features of the Cape Mendocino region affects population structure on a species-by-species basis.

Our current analysis is limited by the unavailability of some species at some locations, and by our focus on a single mtDNA region for comparison. Further work on the fine scale geography of differentiation using COI and other loci is warranted in order to discern the causes of different levels of dispersal interruption among species.

### Pelagic period and genetic structure

Across the 50 species studied here, we find that species without a pelagic period have the highest structure, in accord with many previous studies [13], [14], [15], [16], [17], [42], [43]. However, if only species with pelagic larvae are considered, duration is uncorrelated with overall population subdivision (Figure 3). Some previous multi-species studies also showed poor relationship between larval duration and genetic structure [21], and several striking genetic clines have been described for species that were expected to have high dispersal [20], [44], [45].

These inconsistencies have gone largely unexplained. Barber et al. [46] suggested that larval settlement behavior might explain the difference in the scale of population genetic differentiation among stomatopod crustacea. Cowen et al. [47] also focused on larval behavior as a major determinant of dispersal in Caribbean fishes. Sivasundar and Palumbi (submitted) showed that *Sebastes* rockfish that differed in settlement behavior had different scales of population structure. Shanks and Eckert [37] suggested that differences in breeding date could help explain dispersal patterns in California fish and crustaceans because of seasonal differences in current direction and strength. These results point to the importance of understanding details of the relevant larval ecology in explaining genetic change among populations. Because larvae are not passive particles, integrating behavioral data, seasonal information and oceanographic models with larval durations is likely to increase the explanatory power of larval life histories and genetic differentiation among species.

### Habitat correlates of genetic structure

Though larval duration provides little explanatory power, we see a marked increase in genetic structure among high-to-mid intertidal species. The species with strong structure include acorn barnacles, intertidal limpets, and shore crabs, as well as several species of snails and a tide pool copepod with low larval dispersal potential (Table 2, and Supplementary Information). The association of habitat with structure is not perfect: many intertidal species have no structure and some subtidal species do.

Marko [16] noted an apparently contrasting trend, with a significantly greater proportion of genetically structured species occurring in the lower midlittoral zone than in the upper midlittoral and higher zones. He attributed this to the effects of Pleistocene glaciation, which would have eliminated the habitat of the upper-intertidal species' northern populations. However, we note several key differences between our results and Marko's. First, the trend we report includes only species with planktonic development, minimizing developmental type as a potentially confounding variable. By contrast, half of the species that Marko considered do not disperse planktonically: looking at just those with planktonic development, his observed trend disappears. Secondly, our northern samples came from Sitka, Alaska, which was likely not glaciated in the latest Pleisocene given the genetic diversity (an in some cases, clade structure) present in those samples, and therefore not subject to the effect Marko identified. Finally, the increased number of species we sampled was designed to be a more robust test of precisely this kind of hypothesis, providing statistical power to discern general trends impacting a larger number of taxa. We included as many species from the published literature as the original authors' geographic sampling would allow (including 3 of 8 species Marko analyzed), and did not observe the same trends in the larger dataset.

Candidate mechanisms for increased genetic structure of high intertidal species include differential larval movement and selection. High intertidal species, immersed for only part of the day, likely have fewer opportunities to launch larvae into the water column and fewer chances for larvae to settle. Alternatively, intertidal species may have larvae that behaviorally remain close to shore, thereby limiting along shore movement. This type of explanation has been offered in the case of west coast rockfish, for which shallow water species appear to have more structure than deeper water species [48]. This possibility is weakened by the observation of larvae of intertidal species such as acorn barnacles in offshore oceanic fronts [49], [50], and the weak genetic differentiation of low intertidal species that are just as tied to shore-based habitats as are upper intertidal species (Figure 4).

It is also possible that greater aerial exposure in the high-mid intertidal zones subjects these species to a greater variety of environmental stresses that may generate selective gradients along latitudinal ranges. Previous work on allozyme variation has shown the power of selection to generate structure along environmental gradients in marine and terrestrial systems by acting on individual loci [51], [52], [53], [54], [55], [56]. Intertidal exposure is tied in the barnacle *Semibalanus balanoides* to variation in allele frequencies at allozymes under strong selection, but does not alter mtDNA frequencies [54].

Along the west coast of North America, latitude, the time of exposure to low tide and the probability of coastal fog all combine to create a patchwork of physiological stresses for high intertidal species [57]. The strongest current tests of selection focus on the tidepool copepod *Tigriopus californicus* [58], where large genetic differences across Point Conception are the exception to the geographic patterns we show here. In this case, mtDNA variation is linked to adaptive interactions between nuclear and mitochondrial gene products. Testing selection in other species with striking genetic differentiation (*Balanus glandula, Lottia digitalis, Lottia pelta, Pagurus spp.*) could reveal important mechanisms of evolution in continuous populations with high dispersal potential across environmental gradients.

### Genetics and dispersal

Genetic differentiation is a signal that demographic connection between populations is limited, but the link between genetic subdivision and the exchange of migrants is not perfect. Selection can generate substantial genetic differentiation in the face of high dispersal, as in estuarine mussels that are selected each year for particular LAP allozyme alleles [59]. In the case of the genetic patterns we report here, mtDNA haplotypes we have observed differ by just a few base pairs in general, and it would be unusual if strong selection was acting across species in the same ways. Nevertheless, if the rocky intertidal species studied here show this type of strong selection at COI, then the genetic clines we describe may not reflect dispersal limits but rather a balance between selection and dispersal [34].

Explaining the lack of structure for species with seemingly modest dispersal requires similar caution. The snail *Tegula funebralis* has a 5-day larval period yet shows practically no structure from Oregon to southern California. Populations of this snail are extremely abundant in low intertidal habitats, and as a result very low *per capita* migration rates might result in moderate or high levels of total gene flow (typically measured as the population size times the per capita migration, *Nm*). In other cases, ecologically rare dispersal may nevertheless be large enough to be evolutionarily important, resulting in genetic differentiation that is indistinguishable from zero because of sampling error [60]. Third, populations may not be at drift-migration equilibrium [61]: recent shifts in populations due to glacial cycles may have obscured genetic differentiation and give the impression of high contemporary larval exchange. These various reasons urge caution in interpreting lack of genetic structure in low dispersal marine species.

### Management implications

The 1999 California Marine Life Protection Act mandates a system of marine protected areas along the California coast to support marine ecosystem diversity and stability. Over the past several years, a statewide process for designing and implementing protected area networks has been based on four broad biotic zones based on the biogeographic boundary at Pt. Conception and three other practical socio-political borders. Cape Mendocino sits squarely within Zone 4 of the MLPA process, yet our data suggest that this area may be a fence that limits larval dispersal and population connectivity (see http://mlpa.dfg.ca.gov).

Such fences may disrupt the connections among protected areas, and greatly reduce their ability of the network to stabilize marine populations [62], [63], [but see 64, suggesting that such regions may be valuable as MPAs because they agglomerate alleles from either side of the metaphorical fence]. A break in dispersal along a coastline could indicate that California MPAs will have only limited influence on marine populations to the north of Cape Mendicino, in Oregon and Washington. These suggestions do not apply to all marine species, because the influence of Cape Mendocino appears to vary from species to species in our data set, but the effects of such cryptic marine barriers are notable for their direct policy implications in California and elsewhere.

Taken together, our findings contribute to a view of marine populations as existing in a complex patchwork of habitats that is often obscured by the habitats' superficial similarity. Rather than a process driven primarily by differences in pelagic larval duration, genetic structure may be also often driven by differences in selection across environmental gradients, and by complex larval adaptations that reduce effective dispersal.

### Methods

Specimens were collected live from the field between 2006 and 2008 and preserved in 95% ethanol at 4°C until they could be processed. The four focal collection locations were chosen to span regions of ecological or geographic interest (Figure 1). The southern region (between Monterey and Santa Barbara, California) spanned Point Conception, a focal point for biogeography and phylogeography because of its associated faunal turnover and an abrupt change in sea surface temperature and current regime [25], [27], [29], [65], [66]. The central region (Monterey to southern Oregon) spanned a gradient in primary productivity and upwelling [67] known to be coincident with at least one marine genetic cline [30, Galindo and Palumbi submitted, Jacobs-Palmer and Palumbi in prep], as well as a strong upwelling zone at Cape Mendocino, California, a prominent coastal feature. The northern region (southern Oregon to Sitka, Alaska) spanned a divergence of sea surface currents near Vancouver Island, British Columbia [68], and the maximum extent of the Pleistocene glaciation, thought to have destroyed much of the intertidal habitat north of ca. 49°N latitude until approximately 12–13kya [69].

In some cases, individual species could not be found at the target collection locations, and nearby sites were sampled instead (all collection locations shown in Table 1). Because species ranges vary, not every species could be sampled at every location.

34 invertebrate species were sampled to form the original dataset included here, averaging 18 individuals per population per species, between 2 and 4 geographic locations sampled for each species (Table 1). In some cases, additional collections were made at higher spatial resolution to investigate local barriers to gene flow; these are also included in Table 1. The remaining 16 species were drawn from the published literature; the overall taxonomic sampling was as follows: crustaceans (n = 21 spp.), molluscs (21 spp.), echinoderms (6 spp.), annelids (1 sp.), cnidarians (1 sp.). We have submitted the sequence data from our original dataset to Genbank, where they will be publicly available.

PCR and subsequent sequencing was carried out on genomic DNA as described in [70]. Genetic data were analyzed with Arlequin software for the Macintosh or Windows [71], [72], used to calculate the overall amount of genetic subdivision among populations (Φ_ST_), pairwise subdivision among populations (Slatkin's Linearized F_ST_), and nucleotide diversity (θ) using a Kimura 2-parameter correction. Genetic data were gathered from the published literature as noted in Table 2, and Supplementary Information, and in three cases for which only gene trees were published, clade-level allelic differences between populations were estimated to result in very high F_ST_ values (ca. 0.5–1.0), though our results are not strongly influenced by the magnitudes of these estimations.

Ecological and life history information for each species was gathered from the primary literature (Table 2, and Supplementary Information). Biotic characters were entered as either numeric (e.g., minimum pelagic larval duration, in days) or categorical (e.g., phylum) independent variables, with measures of genetic subdivision (pairwise population F_ST_ and overall Φ_ST_) used as numeric dependent variables for single or multiple linear regression or ANOVA. In total, 22 biotic characters were evaluated for significant correlations with genetic subdivision. Tajima's D calculated using the Bioinformatics toolbox for Matlab (Mathworks, Inc.). All statistical analyses were carried out using the free software package R (http://www.R-project.org).

Because the taxa sampled were spread widely across the animal kingdom, no reliable species-level phylogeny was available for a phylogenetic independent contrasts test of the explanatory variables included in the analysis [see 73], [74]. We therefore accounted for phylogenetic nonindependence by relying on taxonomic grouping as a proxy for phylogeny, looking for confounding effects in two ways: first, we tested for correlations between relevant ecological variables and taxonomic identity at the phylum, order, and family levels. Where significant phylogenetic effects were observed, they are noted. Second, we subsampled the dataset so that only one species from each taxonomic group (family or order) was present. We then used 100 replicate subsampled datasets to assess the distribution of determination coefficients between relevant variables; this process ensured that the observed significant correlations were not artifacts of nonuniform sampling across taxonomic groups.

## Supporting Information

Supplementary Information S1 Table of ecological and life history information used for analysis. Genetic results in bold are significantly different from zero. Larval types were sorted into practical categories; while some crustacean groups have different names for terminal larval stages, those that reasonably approximated megalopae were labeled as such. Larval trophic level was treated similarly, sorting groups into functional categories. Habitat depth was treated in a variety of different ways, the most relevant presented here: divided into seven categories, four categories, and an ordered set of seven numerical values. Pairwise FST calculations are shown for pairs of populations: “Mont-OR,” for example, is the pairwise FST between Monterey and Oregon. SB  =  Santa Barbara, Mont  =  Monterey, OR  =  Oregon, AK  =  Alaska. SAShA OM represents the mean geographic distance between shared alleles; SAShA OM/Exp is the ratio of the observed mean geographic distance between alleles to the expected distance given sampling (see Kelly et al., Journal of Heredity, in press). Tajima's D was calculated for the overall sample of all populations combined, with data trimmed to remove missing characters.
(0.06 MB PDF)
