# Supplementary material for: Genetic Structure Among 50 Species of the Northeastern Pacific Rocky Intertidal Community
Source: PLoS One. 2010 Jan 7;5(1):e8594. doi: 10.1371/journal.pone.0008594 (PMC2799524; doi:10.1371/journal.pone.0008594)
Supplement: Supplementary Information S1 — Table of ecological and life history information used for analysis. Genetic results in bold are significantly different from zero. Larval types were sorted into practical categories; while some crustacean groups have different names for terminal larval stages, those that reasonably approximated megalopae were labeled as such. Larval trophic level was treated similarly, sorting groups into functional categories. Habitat depth was treated in a variety of different ways, the most relevant presented here: divided into seven categories, four categories, and an ordered set of seven numerical values. Pairwise FST calculations are shown for pairs of populations: “Mont-OR,” for example, is the pairwise FST between Monterey and Oregon. SB = Santa Barbara, Mont = Monterey, OR = Oregon, AK = Alaska. SAShA OM represents the mean geographic distance between shared alleles; SAShA OM/Exp is the ratio of the observed mean geographic distance between alleles to the expected distance given sampling (see Kelly et al., Journal of Heredity, in press). Tajima's D was calculated for the overall sample of all populations combined, with data trimmed to remove missing characters. (0.06 MB PDF) [file pone.0008594.s001.pdf]

Species	Phylum	Order	Family	Min. PLD	Larval Type	Larval Trophic Level	Fertilization Type	Min. Egg Size (um)	Adult Trophic Level	Tidal Height (7 categories)	Tidal Height (4 categories)	Tidal Code (ordered)	Min. Latitude (Deg)	Midpoint Lat. (Deg)	Max. Latitude (Deg)	Lat. Range (Deg)	Habitat	Max Size (mm)	COI Nucleotide Diversity	PhiST	SB-Mont FST	Mont-OR FST	OR-AK FST	SAShA OM (km)	SAShA OM/Exp (km)	Tajima's D
Phragmatopoma californica	Annelida	Flabelligerida	Sabellariidae	18	trochophore	primary consumer	spawns	75	primary consumer	mid-intertidal	mid	5	31.8	34.8	37.8	6	Rock Intertidal	50	0.011328	0.01978	0.01978	NA	NA	141.4286	0.834821423	-1.554289
Balanus glandula	Arthropoda	Thoracicia	Balanidae	14	cyprid	primary consumer	internal	100	primary consumer	high intertidal	high	7	27.8	43.4	59	31.2	Rock Intertidal	22	NA	NA	**0.043**	**0.329**	NA	NA	NA	NA
Cancer antennarius	Arthropoda	Decapoda	Cancridae	60	megalopa	secondary consumer	pseudointernal	333	secondary consumer	low intertidal-subtidal	low	2	23.2	36.6	50	26.8	Rock Subtidal	118	NA	NA	NA	NA	NA	167.0245	1.027843077	-1.599382
Cancer productus	Arthropoda	Decapoda	Cancridae	100	megalopa	secondary consumer	pseudointernal	367	secondary consumer	low intertidal-subtidal	low	2	32.7	45.85	59	26.3	Rock Subtidal	158	NA	NA	NA	NA	NA	607.7835	0.884989455	-0.958462
Emerita analoga	Arthropoda	Decapoda	Hippidae	70	megalopa	secondary consumer	pseudointernal	110	primary consumer	low intertidal-subtidal	low	2	31.8	45.4	59	27.2	Sand or Mud	35	0.00386	-0.01509	-0.00713	-0.01681	NA	500.6215	0.982942961	-2.292866
Hemigrapsus nudus	Arthropoda	Decapoda	Grapsidae	30	megalopa	secondary consumer	pseudointernal	380	primary consumer	high-mid	high	6	34.5	46.75	59	24.5	Rock Intertidal	56	0.00792675	**0.43829**	NA	**0.02432**	**0.59492**	377.5611	0.396115299	-1.571543
Idotea cf. stenops	Arthropoda	Isopoda	Idoteidae	0	crawl-away	primary consumer	internal	NA	primary consumer	low intertidal	low	3	33	35.1	37.2	4.2	On AlgaePlants	10	0.000907	**0.8607**	**0.8607**	NA	NA	0	0	-0.384201
Idotea kirchanskii	Arthropoda	Isopoda	Idoteidae	0	crawl-away	primary consumer	internal	NA	primary consumer	low intertidal	low	3	33	35.1	37.2	4.2	On AlgaePlants	15	0.009335	**0.14527**	**0.14527**	NA	NA	151.2524	0.726621301	-1.37666228
Idotea montereyensis	Arthropoda	Isopoda	Idoteidae	0	crawl-away	primary consumer	internal	NA	primary consumer	low intertidal	low	3	35.3	47.15	59	23.7	On AlgaePlants	16	0.009	**0.08718**	NA	**0.13337**	-0.01259	328.7059	0.290313789	-0.22070081
Lophopanopeus bellus	Arthropoda	Decapoda	Cancridae	30	megalopa	secondary consumer	pseudointernal	330	omnivore	low intertidal-subtidal	low	2	35.3	47.15	59	23.7	Rock Intertidal	34	0.00594267	0.00727	NA	0.03239	0.00154	1025.2	1.077455402	-2.414855
Pachygrapsus crassipes	Arthropoda	Decapoda	Grapsidae	30	megalopa	secondary consumer	pseudointernal	NA	primary consumer	high intertidal	high	7	28	43.5	59	31	Rock Intertidal	47	0.009	NA	0.0034	-0.00055	-0.00055	NA	NA	NA
Pagurus granosimanus	Arthropoda	Decapoda	Paguroidea	70	megalopa	secondary consumer	pseudointernal	NA	detritivore	low intertidal-subtidal	mid	2	31.8	45.4	59	27.2	Rock Intertidal	19	0.010859	**0.1032**	-0.00797	**0.04453**	**0.5**	341.3077	0.420929712	-1.990453
Pagurus hirsutiusculus	Arthropoda	Decapoda	Paguroidea	67	megalopa	secondary consumer	pseudointernal	NA	detritivore	mid-intertidal	mid	5	36.5	47.75	59	22.5	Rock Intertidal	19	0.0027	**0.35744**	NA	**0.08365**	**0.8331**	276.1722	0.537478079	-1.619175
Pagurus samuelis	Arthropoda	Decapoda	Paguroidea	51	megalopa	secondary consumer	pseudointernal	NA	detritivore	mid-intertidal	mid	5	27.85	43.425	59	31.15	Rock Intertidal	19	0.005562	**0.10449**	**0.11933**	**0.04867**	NA	281.8058	0.927166912	-2.044343
Pagurus venturensis	Arthropoda	Decapoda	Paguroidea	50	megalopa	secondary consumer	pseudointernal	NA	detritivore	mid-intertidal	mid	4	28	32.6	37.2	9.2	Rock Intertidal	19	0.0073	**0.25245**	NA	NA	NA	NA	NA	1.384492
Pandalus platyceros	Arthropoda	Decapoda	Pandalidae	150	megalopa	secondary consumer	pseudointernal	2000	secondary consumer	deep water	deep water	0	32.7	45.85	59	26.3	Rock Subtidal	254	0.0037615	-0.00616	-0.00616	NA	NA	172.8571	1.018860373	-0.188281
Petrolisthes cinctipes	Arthropoda	Decapoda	Porcellanidae	30	megalopa	secondary consumer	pseudointernal	800	detritivore	high-mid	mid	6	34.5	44.1	53.7	19.2	Rock Intertidal	24		0.01456	NA	0.01456	NA	280.875	0.818181739	-1.351886
Pollicipes polymerus	Arthropoda	Thoracicia	Scalpellidae	42	cyprid	primary consumer	internal	100	primary consumer	high-mid	mid	6	27.85	42.375	56.9	29.05	Rock Intertidal	80	0.00616	**0.03356**	**0.09881**	**0.05929**	0.03261	559.6337	0.734010236	-2.171836
Pugettia gracilis	Arthropoda	Decapoda	Majidae	120	megalopa	secondary consumer	pseudointernal	NA	primary consumer	low intertidal-subtidal	low	2	36.5	47.75	59	22.5	Rock Intertidal	39	0.001	0.00252	NA	NA	0.00252	876.375	1.057031262	-1.88235
Semibalanus cariosus	Arthropoda	Thoracicia	Archaeobalanidae	90	cyprid	primary consumer	internal	NA	primary consumer	mid-low	mid	4	35.3	47.15	59	23.7	Rock Intertidal	60	0.004	-0.00057	NA	0.00657	0.00081	887.7789	0.908292319	-2.146808
Tetraclita squamosa	Arthropoda	Thoracicia	Tetraclitidae	20	cyprid	primary consumer	internal	290	primary consumer	high-mid	high	6	26	31.75	37.5	11.5	Rock Intertidal	30	NA	0.011	0.011	NA	NA	NA	NA	NA
Tigriopus californicus	Arthropoda	Harpactacoida	Harpactacicidae	28	megalopa	secondary consumer	pseudointernal	100	primary consumer	high intertidal	high	7	28	43.5	59	31	Rock Intertidal	1.4	NA	**0.98**	**0.98**	**0.98**	**0.98**	NA	NA	NA
Anthopleura elegantissima	Cnidaria	Actiniatia	Actiniidae	30	planula	primary consumer	spawns	135	omnivore	mid-low	mid	4	31.8	45.4	59	27.2	Rock Intertidal	250	0	0	NA	NA	NA	482.9271	1	NA
Cucumaria pseudocurata	Echinodermata	Dendrochirotida	Cucumariidae	0	pluteus	nonfeeding	spawns	916	primary consumer	low intertidal-subtidal	low	2	36.5	47.75	59	22.5	Rock Intertidal	35	NA	**0.5**	NA	**0.5**	NA	NA	NA	NA
Parastichopus parvimensis	Echinodermata	Aspidochirotida	Stichopodidae	50	pluteus	primary consumer	spawns	NA	detritivore	low intertidal-subtidal	low	2	27.8	32.15	36.5	8.7	Sand or Mud	250	NA	0.0001	0.0001	NA	NA	NA	NA	NA
Pisaster giganteus	Echinodermata	Forcipulatida	Asteriidae	60	pluteus	primary consumer	spawns	165	secondary consumer	low intertidal-subtidal	low	2	27.85	38.925	50	22.15	Rock Intertidal	300	0.001446	-0.02613	-0.02613	NA	NA	163.1377	0.997386363	-1.025472
Pisaster ochraceus	Echinodermata	Forcipulatida	Asteriidae	76	pluteus	primary consumer	spawns	150	secondary consumer	mid-low	mid	4	34.5	46.75	59	24.5	Rock Intertidal	140	0.00379374	NA	0	**0.03**	NA	NA	NA	NA
Pycnopodia helianthoides	Echinodermata	Forcipulatida	Asteriidae	70	pluteus	primary consumer	spawns	120	secondary consumer	low intertidal-subtidal	low	2	31.8	45.4	59	27.2	Rock Subtidal	900	0.0018506	**0.13721**	0.00353	NA	-0.07797	977.7655	0.764118084	-1.563831
Strongylocentrotus franciscanus	Echinodermata	Echinoidea	Strongylocentrotidae	70	pluteus	primary consumer	spawns	130	primary consumer	low intertidal-subtidal	low	2	27.8	43.4	59	31.2	Rock Subtidal	100	0.000954	0.009	0.03155	NA	NA	1495.8	1.065081173	-1.806017
Acanthina spirata	Mollusca	Neogastropoda	Muricidae	0	crawl-away	nonfeeding	internal	NA	secondary consumer	high intertidal	high	7	29.9	34.05	38.2	8.3	Rock Intertidal	40	NA	**0.5**	0.5	NA	NA	NA	NA	NA
Alderia modesta	Mollusca	Sacoglossa	Stiligeridae	35	veliger	primary consumer	internal	78	primary consumer	low intertidal	low	3	37.5	48.25	59	21.5	Sand or Mud	4	0.0209	0.00838	NA	-0.01151	-0.00143	NA	NA	NA
Alderia willowi	Mollusca	Sacoglossa	Stiligeridae	2	veliger	nonfeeding	internal	105	primary consumer	low intertidal	low	3	32	34.75	37.5	5.5	Sand or Mud	2.4	0.0141	0.01197	-0.00926	NA	NA	NA	NA	NA
Aplysia californica	Mollusca	Anaspidea	Aplysiidae	30	veliger	primary consumer	internal	NA	primary consumer	low intertidal-subtidal	low	2	30	35	40	10	Sand or Mud	400	NA	0.0084	0.0084	NA	NA	NA	NA	NA
Calliostoma ligatum	Mollusca	Archaegastropoda	Trochidae	7	veliger	nonfeeding	spawns	220	primary consumer	mid-low	low	4	32.7	45.85	59	26.3	Rock Intertidal	33	0.000933	-0.00898	NA	-0.00814	0.00192	935.8144	1.037763221	-2.345953
Cyanoplax dentiens	Mollusca	Neoloricata	Lepidochitonidae	6	veliger	nonfeeding	spawns	200	primary consumer	mid-intertidal	mid	5	34.5	46.75	59	24.5	Rock Intertidal	20	0.0125752	**0.03871**	NA	**0.06542**	NA	NA	NA	NA
Fissurella volcano	Mollusca	Archaegastropoda	Fissurellidae	4	veliger	nonfeeding	spawns	205	primary consumer	mid-intertidal	mid	5	31.8	36.8	41.8	10	Rock Intertidal	35	0.00058	0.01089	0.01089	NA	NA	166.25	0.986032443	-1.497566
Haliotis rufescens	Mollusca	Archaeogastropoda	Haliotidae	4	veliger	nonfeeding	spawns	NA	primary consumer	low intertidal-subtidal	low	2	28	35	42	14	Rock Subtidal	314	NA	0.007	-0.008	0.017	NA	NA	NA	NA
Katharina tunicata	Mollusca	Neoloricata	Mopaliidae	7	veliger	nonfeeding	spawns	230	primary consumer	mid-low	mid	4	34.5	46.75	59	24.5	Rock Intertidal	130	0.00904567	0.02932	NA	-0.00952	0.07578	1062.3529	1.165150263	-1.849609
Lottia austrodigitalis	Mollusca	Patellogastropoda	Lottiidae	5	veliger	nonfeeding	spawns	NA	primary consumer	high intertidal	high	7	23.2	29.85	36.5	13.3	Rock Intertidal	26	0.0015	-0.03802	-0.03802	NA	NA	172.0623	1.020132012	-1.416259
Lottia digitalis	Mollusca	Patellogastropoda	Lottiidae	5	veliger	nonfeeding	spawns	200	primary consumer	high intertidal	high	7	36.5	47.75	59	22.5	Rock Intertidal	26	0.001	**0.61082**	NA	**0.64658**	0.01288	441.9937	0.667022165	0.722903
Lottia new sp. cf pelta	Mollusca	Patellogastropoda	Lottiidae	5	veliger	nonfeeding	spawns	NA	primary consumer	mid-low	mid	4	27.85	32.175	36.5	8.65	Rock Intertidal	40	0.003	-0.03298	-0.03298	NA	NA	136.6738	0.880815739	0.641611
Lottia paradigitalis	Mollusca	Patellogastropoda	Lottiidae	5	veliger	nonfeeding	spawns	NA	primary consumer	high-mid	mid	6	36.5	47.75	59	22.5	Rock Intertidal	10	0.00083225	0.00055	0.0288	-0.03858	-0.03255	931.4729	1.033978304	-1.274904
Lottia pelta	Mollusca	Patellogastropoda	Lottiidae	5	veliger	nonfeeding	spawns	136	primary consumer	mid-low	mid	4	36.5	47.75	59	22.5	Rock Intertidal	40	0.001416	**0.56145**	NA	-0.00683	**0.67274**	357.9574	0.343760108	-0.863045
Macoma nasuta	Mollusca	Veneroida	Tellinidae	35	veliger	primary consumer	spawns	NA	primary consumer	low intertidal-subtidal	low	2	23.2	41.1	59	35.8	Sand or Mud	110	NA	0.01147	-0.00616	0.0116	NA	491.3358	1.005985104	-2.359047
Mytilus californianus	Mollusca	Mytiloida	Mytilidae	9	veliger	primary consumer	spawns	60	primary consumer	mid-intertidal	mid	5	27.8	43.4	59	31.2	Rock Intertidal	130	NA	-0.00096	0	0	0	NA	NA	NA
Nucella emarginata	Mollusca	Neogastropoda	Muricidae	0	crawl-away	nonfeeding	internal	180	secondary consumer	high-mid	high	6	31.8	34.4	37	5.2	Rock Intertidal	40	NA	**0.5**	**0.7**	NA	NA	NA	NA	NA
Nucella ostrina	Mollusca	Neogastropoda	Muricidae	0	crawl-away	nonfeeding	internal	NA	secondary consumer	high-mid	high	6	34.5	46.75	59	24.5	Rock Intertidal	40	NA	**0.2**	NA	**0.7**	0	NA	NA	NA
Olivella biplicata	Mollusca	Neogastropoda	Olividae	1	veliger	nonfeeding	internal	NA	omnivore	low intertidal-subtidal	low	2	31.8	40.9	50	18.2	Sand or Mud	30	0.0065	0.00621	0.00025	0.01235	NA	519.975	0.973170641	-2.121162
Lirabuccinum (Searlesia) dira	Mollusca	Neogastropoda	Buccinidae	0	crawl-away	nonfeeding	internal	240	secondary consumer	mid-low	mid	4	36.5	47.75	59	22.5	Rock Intertidal	45	0	**1**	NA	NA	**1**	0	0	2.610901
Tegula (Chlorostoma) funebralis	Mollusca	Archaegastropoda	Trochidae	5	veliger	nonfeeding	spawns	30	primary consumer	mid-intertidal	mid	5	31.8	40.9	50	18.2	Rock Intertidal	30	0.007083	0.01674	-0.00047	0.01276	NA	441.1862	0.961656141	-2.413437
